# The Anti-Amoebic Activity of a Peptidomimetic against *Acanthamoeba castellanii*

**DOI:** 10.3390/microorganisms10122377

**Published:** 2022-11-30

**Authors:** Hari Kumar Peguda, Nicole A. Carnt, Zi Gu, Naresh Kumar, Mark D. P. Willcox, Rajesh Kuppusamy

**Affiliations:** 1 School of Optometry and Vision Science, University of New South Wales, Sydney 2052, Australia; 2 School of Chemical Engineering, University of New South Wales, Sydney 2052, Australia; 3 School of Chemistry, University of New South Wales, Sydney 2052, Australia

**Keywords:** *Acanthamoeba*, free-living amoeba, anti-microbial peptides, peptidomimetics

## Abstract

*Acanthamoeba* is a free-living protozoan known to cause keratitis most commonly, especially among contact lens wearers. Treatment of *Acanthamoeba* keratitis is challenging as *Acanthamoeba* can encyst from the active form, a trophozoite, into a hibernating cyst that is refractory to antibiotics and difficult to kill; therefore, there is a need for more effective anti-amoebic strategies. In this study, we have evaluated the anti-amoebic activity of the antimicrobial peptide mimic RK-758 against *Acanthamoeba castellanii*. RK-758 peptidomimetic was subjected to biological assays to investigate its amoebicidal, amoebistatic, anti-encystation, and anti-excystation effects on *A. castellanii*. The anti-amoebic activity of the peptide mimic RK-758 was compared with chlorhexidine against the *Acanthamoeba castellanii* ATCC30868 and *Acanthamoeba castellanii* 044 (a clinical strain) with the concentrations of both ranging from 125 µM down to 7.81 µM. All experiments were performed in duplicate with three independent replicates. The data were represented as mean ± SE and analysed using a two-sample *t*-test and two-tailed distributions. A *p* < 0.05 was considered statistically significant. The peptidomimetic RK-758 had anti-*Acanthamoeba* activity against both trophozoites and cysts in a dose-dependent manner. The RK-758 had amoebicidal and growth inhibitory activities of ≥50% at a concentration between 125 µM and 15.6 µM against the trophozoites of both *Acanthamoeba* strains. Inhibitory effects on the cyst formation and trophozoite re-emergence from cysts were noted at similar concentrations. Chlorhexidine had 50% activity at 7.81 µM and above against the trophozoites and cysts of both strains. In the haemolysis assay, the RK-758 lysed horse RBCs at concentrations greater than 50 µM whereas lysis occurred at concentrations greater than 125 µM for the chlorhexidine. The peptidomimetic RK-758, therefore, has activity against both the trophozoite and cyst forms of *Acanthamoeba* and has the potential to be further developed as an anti-microbial agent against *Acanthamoeba*. RK-758 may also have use as an anti-amoebic disinfectant in contact lens solutions.

## 1. Introduction

*Acanthamoeba* is a single-celled eukaryotic microorganism known to cause rare but sight-threatening keratitis, especially among contact lens (CL) wearers [1,2]. *Acanthamoeba* exists in two forms, the trophozoite is the pathogenic form which is metabolically active and responsible for causing the corneal infection, whereas the cyst form is metabolically dormant or inactive but resistant to disinfectants and antimicrobials [3]. The phenotypic switching between the trophozoite and cyst stages makes it difficult to diagnose and treat this infection [4]. Even if it is diagnosed early, the prognosis is poor due to a lack of effective treatment modalities. The treatment of *Acanthamoeba* keratitis (AK) remains a challenge and it can lead to corneal toxicity. The current treatment mainly involves topical medications such as chlorhexidine (0.02–0.2%) or polyhexamethylene biguanide (PHMB) alone or in combination with diamidines (propamidine isethionate or hexamidine (0.1% *w*/*v*), oral miltefosine, and penetrating keratoplasty in non-responding cases [3,5,6,7]. Furthermore, the therapeutic agents need to be given to patients as topical drops hourly (day and night) for at least the first few days after diagnosis [8]. The treatment of AK may continue for up to 6 months to 1 year or even longer [4,9]. Topical biguanides are the commonly used agents as they can be active against cysts in vivo, unlike many other drugs which have limited cysticidal activity [9]. Even then, *Acanthamoeba* can be resistant to these normal treatment modalities, allowing the disease to worsen [5]; therefore, there is a need for better and more effective treatment options for this disease.

Antimicrobial peptides (AMPs) are naturally occurring molecules that have gained importance due to their broad-spectrum antimicrobial properties [10]. AMPs have the advantage that it is difficult for microorganisms to develop resistance to them [11]. A total of 3425 antimicrobial peptides have been registered and stored on the antimicrobial database (APD3) as at June 2022 [12,13]. These AMPs can be classified into 18 categories based on their activities [11]. Among them, the majority (85%) are antibacterial peptides, followed by 37% showing antifungal properties, with some of the remainder being potentially useful to treat cancer, having antiviral properties. Antiparasitic AMPs account for approximately 4% (140 out of 3425) [12].

Most AMPs are highly cationic and act by membrane disruption with selective binding to the negatively charged surfaces of pathogens. The most membrane-active AMPs contain both hydrophilic and hydrophobic groups which aggregate at the cell membranes after initial electrostatic interactions with negatively charged lipid groups in prokaryotic membranes, followed by insertion into the cell membrane using their hydrophobic meioties [14]. Cationic AMPs may have a reduced affinity towards eukaryotic cells as these cells generally possess a neutral charge. AMPs are effective against eukaryotic microbes such as yeasts (e.g., *Candida* sp., *Cryptococcus* sp., and *Saccharomyces* sp.) and protozoa (*Leishmania* sp.). For example, histatin 5 is active against *Candida* sp. and *Aspergillus fumigatus* [15], and dermaseptin is active against *Leishmania major*, *Cryptococcus neoformans*, and *Plasmodium falciparum* [16]; however, these naturally occurring AMPs can be difficult to synthesize in copious quantities and they are susceptible to proteolytic degradation [17,18].

The highly cationic peptide, protamine, its derivative, melimine, and magainin are active against *Acanthamoeba* [10,19,20,21]. Magainin combined with silver nitrate or other antimicrobial agents further enhances the activity against *Acanthamoeba* trophozoites and cysts [20]. Ceragenin, a cationic steroid antibiotic (CSA)-13 that mimics the activity of endogenous AMPs showed amoebicidal activity in a dose-dependent manner [22]. Additionally, α-helical and β- sheeted AMPs based on trialysin and gomesin, respectively, can permeabilise *A. castellanii* but these peptides were sensitive to the proteases released by trophozoites [23]. Another α-helical peptide, Ci-MAM-A24, showed activity against *A. castellanii* by permeabilising the cell membrane at relatively higher concentrations. This AMP was also able to reduce the number of *Legionella* residing within the *Acanthamoeba* [24]. Poly-epsilon-lysine (pεK) peptide, alone or covalently attached to hydrogel contact lenses, is active against *Acanthamoeba* trophozoites and cysts in a phosphate buffer saline or ex vivo corneas [25]. AMPs derived from the antibiotic tyrocidine can have amoebicidal activity and are able to inhibit the encystation of *A. castellanii* and *Naegleria floweri* [26]. A synthetic decapeptide (KP) AMP had time-dependent amoebicidal activity against a *A. castellanii* clinical isolate [27]. Nisin, a natural AMP produced by *Lactococcus lactis*, can also be active against *A. castellanii* trophozoites at the end of a 24 h incubation [28]. Moreover, human corneal limbal cells have shown significant upregulation in the gene expression for seven out of eight AMPs studied after exposure to *A castellanii* trophozoites, indicating a possible role in combating amoebic infection [29].

Peptidomimetics are synthetic compounds designed to mimic the biological function of peptides and overcome the limitations of AMPs, such as their relatively short half-lives in vivo that occurs due to proteolytic degradation, the high cost of their synthesis, and the toxicity of certain AMPs. To mimic the biological function of natural AMPs, peptidomimetic molecules contain amphiphilic, i.e., hydrophobic and hydrophilic backbones with a net positive charge [14]. Studies have shown that these peptidomimetics have an increased stability to enzymatic degradation, improved bioavailability, potent activity against multi-drug resistant bacteria, and synthetic flexibility [30]. Peptidomimetic antibiotic 10, developed from human α- defensin 5, has improved killing activity against Gram-positive and Gram-negative bacteria, including the multi-drug-resistant strains isolated from patients [31]. Novel pyridine-cysteine containing cyclic peptidomimetics have shown high activity against *Candida albicans* and Gram-negative bacteria such as *Pseudomonas aeruginosa*, *Klebsiella pneumoniae*, and *Proteus vulgaris* [32]. Benzodiazepine-based peptidomimetics had activity against the protozoa, *Trypanosoma brucei brucei*, which causes sleeping sickness in humans [33]. The current authors have developed various peptidomimetics which are active against antibiotic-resistant bacteria such as *Staphylococcus aureus*, *Escherichia coli* [34], and *Pseudomonas aeruginosa* [35]. Similarly, cholic acid-based AMPs also exhibited high anti-bacterial potency against Gram-negative and Gram-positive bacteria [36]. Peptide mimics of cathelicidin are highly cationic and have shown an efficient and fast (<30 min) killing of methicillin-susceptible *S. aureus* [37].

Any potential therapeutics for *Acanthamoeba* must not cause the amoeba to encyst, as this may result in a re-activation/re-infection after the cessation of therapy. The importance of encystment was demonstrated in an *Acanthamoeba* keratitis worldwide outbreak. This outbreak occurred as the result of contact lens wearers using Complete MoisturePlus (Advance Medical Optics, Santa Ana, California) as a disinfecting solution [38,39]. There was an independent association seen between AK among soft contact lens users and the complete MoisturePlus multi-purpose solution [40]. It was subsequently found, after the contact lens disinfecting solution had been recalled from sale, that a possible reason why this disinfecting solution was associated with the *Acanthamoeba* keratitis outbreak was that the solution, unlike other disinfectants, caused *Acanthamoeba* to encyst [41]. A case-control study on AK cases between 2005–2007 in the USA highlighted the importance of promoting healthy contact lens handling, safe hygiene practices among new contact lens users and emphasised the need for standardised anti-*Acanthamoeba* testing of contact lens solutions [39]. Similarly, the UK had an AK outbreak between 2010–2011 resulting in three times higher incidence rates than between 2004–2009 [2]. Some of the risk factors identified in this outbreak were an oxipol disinfection, contact lenses made of group IV hydrogel contact lens materials (i.e., high water content, ionic hydrogel lenses), poor CL hygiene practice, no or improper hand washing before CL handling, and swimming with contact lenses [2,42]. In the AK infections associated with overnight orthokeratology lenses, the risk factors identified were the use of tap water to clean lenses and cases, the use of homemade saline, and the use of disinfecting solution added to the previously-used remnants of solution in contact lens cases [43,44].

This study evaluated the anti-amoebic activity of an antimicrobial peptidomimetic, RK-758, against *Acanthamoeba* trophozoites and cysts in comparison with chlorhexidine.

## 2. Materials and Methods

### 2.1. Acanthamoeba Culturing and Test Compounds

The *Acanthamoeba castellanii* strains ATCC30868 and 044 (a clinical isolate) [19] were cultured in a 10 mL protease-peptone yeast glucose (PYG) medium (protease peptone 20 g/L, yeast extract 2 g/L and glucose 18 g/L without additives) [10] in 75 cm^2^ tissue culture flasks at 32 °C for 5–7 days. Trophozoites were collected once a 90% confluency was achieved by washing and resuspending in 1× phosphate buffered saline (PBS; NaCl 8 g/L, KCL 0.2 g/L, Na_2_HPO4 1.4 g/L, KH_2_PO_4_ 0.24 g/L, and pH 7.2) or PYG (depending upon the subsequent assay) by centrifugation at 500 g for 2 min. Cysts were obtained by seeding approximately 5 × 10^5^ trophozoites/mL on non-nutrient agar (NNA) plates (NNA: NaCl 0.012 g/L, MgSO_4_. 7H_2_O 0.0004 g/L, CaCl_2_.6H_2_O 0.0004 g/L, Na_2_HPO_4_ 0.0142 g/L, KH_2_PO_4_ 0.0136 g/L, agar 15 g/L, and pH 6.8) and incubating the plates at 32 °C for 14 days. The cysts were scrapped and washed in PBS by centrifugation at 3500× *g* for 10 min and resuspended in PBS [45]. The cysts were stored at 4 °C for a maximum of 14 days.

The test compound in this study, peptidomimetic RK-758, was synthesized according to the patents WO2018081869A1 and Australian Provisional Patent Application No. 2021902457, and its chemical structure has been published [35]. The chlorhexidine was sourced from Sigma Aldrich (St. Louis, MO, USA).

### 2.2. Amoebicidal Assay

Briefly, 5 × 10^5^ trophozoites/mL were incubated with the peptidomimetic RK-758 or chlorhexidine in 24-well plates at concentrations ranging from 125 µM to 7.81 µM in PBS [46]. The chlorhexidine was used as a positive control [47]. PBS alone was used as a negative control. The trophozoites were incubated at 30 °C for 24 h. The number of viable trophozoites was determined by adding 0.1% trypan blue to each well. Dead trophozoites that stained blue and live trophozoites that remained unstained were counted using a Neubauer haemocytometer (Hirschmann, Germany) [46].

### 2.3. Amoebistatic Assay

Briefly, 2 × 10^5^ trophozoites/mL were incubated with the peptidomimetic RK-758 or chlorhexidine in 24-well plates at concentrations ranging from 125 µM to 7.81 µM in PYG [46]. The PYG medium alone was used as a control. After incubation at 30 °C for 48 h, the number of viable trophozoites was determined using a Neubauer haemocytometer after the addition of 0.1% of trypan blue to each well as mentioned.

### 2.4. Encystation Assay

To measure the ability of the peptidomimetic RK-758 or chlorhexidine compared to the ability of the trophozoites to encyst, an encystment medium was prepared by adding 50 mM MgCl_2_ and 10% glucose to 1× PBS by filter sterilization using 0.22 µM membrane filters (Merck, Dublin, Ireland) [48]. The trophozoites (5 × 10^5^ trophozoites/mL) were incubated with either peptidomimetic RK-758 or chlorhexidine in 24-well plates with concentrations ranging from 125 µM to 7.81 µM in the encystment media. The encystment medium alone was used as a control. The trophozoites were incubated at 30 °C for 72 h followed by adding 0.25% (*w*/*v*) sodium-dodecyl sulphate to each well to burst the trophozoites leaving the cysts intact [41]. The number of cysts was determined by counting on a Neubauer haemocytometer.

### 2.5. Excystment Assay

In this assay [45,48], 5 × 10^5^ cysts/mL were incubated with the peptidomimetic RK-758 or chlorhexidine in 24-well plates at concentrations ranging from 125 µM to 7.81 µM in PYG. The PYG medium alone was used as a control. The plates were observed every day at a 10× and 40× magnification to assess the emergence of trophozoites during incubation at 30 °C for 72 h. At the end of the incubation period, the number of trophozoites that re-emerged was counted using a Neubauer haemocytometer.

### 2.6. Lysis of Horse Red Blood Cells (RBCs)

The haemolytic activities of both the chlorhexidine and peptidomimetic RK-758 were determined using horse red blood cells (HRBCs; Oxid, Australia) as described previously [49,50]. Briefly, the HRBCs were washed three times with PBS at 470× *g* for 5 min. Chlorhexidine and peptidomimetic RK-758 concentrations ranging from 200 µM to 12.5 µM were added to the washed HRBCs and incubated at 37 °C for 4 h. The PBS was used as a negative control to achieve a 0% lysis. HRBCs in distilled water were used as positive controls to achieve 100% lysis. At the end of the incubation, the cells were pelleted at 1057× *g* for 5 min, and the supernatant was removed to assess the release of haemoglobin by measuring the OD at 540 nm. The relative OD of the HRBCs treated with the compounds, chlorhexidine and peptidomimetic RK-758, was compared to that of those treated with distilled water and used to determine the relative percentage of haemolysis as follows:

% Haemolysis = (absorbance of test compound) − (absorbance of diluent)

/(absorbance of positive control) − (absorbance of diluent) × 100

### 2.7. Statistical Analyses

The statistical analyses were performed using the GraphPad Prism 8.4.3 software (GraphPad Software, La Jolla, CA, USA). All the experiments were performed in duplicate with three independent replicates. The data were represented as mean ± SE and analysed using a two-sample *t*-test and two-tailed distributions. A *p* < 0.05 was considered statistically significant.

## 3. Results

### 3.1. Amoebicidal Assay

**Figure 1:**
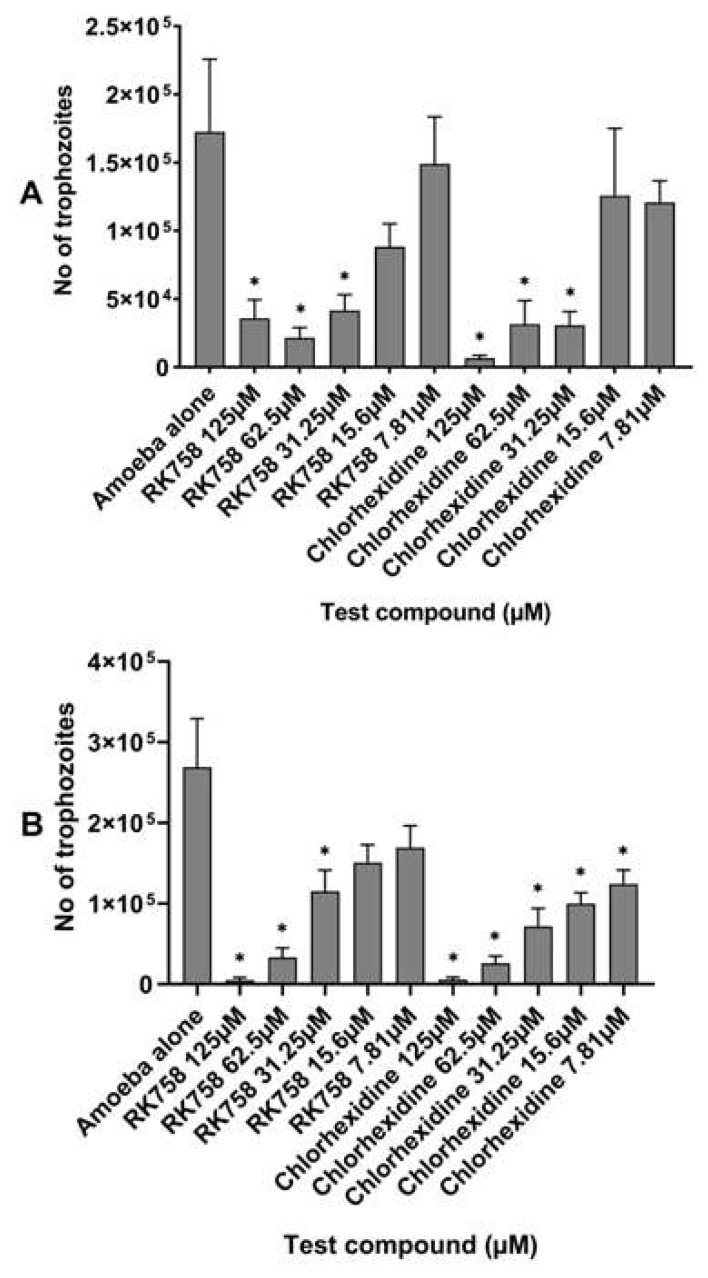
Amoebicidal activity of peptidomimetic RK-758 in comparison with chlorhexidine against *Acanthamoeba castellanii* ATCC30868 (**A**) and *A. castellanii* 044 (**B**). In brief, 5 × 10^5^ *A. castellanii* trophozoites were incubated with the peptidomimetics RK-758 and chlorhexidine at 30 °C for 24 h after which the viability was determined by staining with trypan blue using a Neubauer haemocytometer. The results show significant anti-*Acanthamoeba* activity when compared to the negative control (amoeba alone). * *p* < 0.05 using a two-sample *t*-test and two-tailed distribution.

Amoebicidal assays were performed to determine the activity of the peptidomimetic RK-758 in comparison to the chlorhexidine on the viability of *A. castellanii* ATCC30868 and 044 strains. The number of viable trophozoites was 1.73 × 10^5^ for *A. castellanii* ATCC30868 and 2.69 × 10^5^ for *A. castellanii* 044 following a 24 h incubation in PBS alone. The RK-758 caused statistically significant killing of trophozoites between 79%, 87%, and 76% against A. *castellanii* ATCC30868 (the number of viable trophozoites reduced to 3.58 × 10^4^ (*p* = 0.03), 2.17 × 10^4^ (*p* = 0.01), and 1.6 × 10^4^ (*p* = 0.03), respectively) (Figure 1A), and 98%, 87%, and 57% against A. *castellanii* 044 (the number of viable trophozoites reduced to 5 × 10^3^ (*p* = 0.001), 3.3 × 10^4^ (*p* = 0.003), and 1.1 × 10^5^ (*p =* 0.01),respectively) (Figure 1B), at test concentrations ranging between 125 µM, 62.5 µM, and 31.25 µM, respectively, when compared to the PBS alone. Similarly, chlorhexidine caused statistically significant killing of trophozoites by 96%, 81.5%, and 82% against A. *castellanii* ATCC30868 (the number of viable trophozoites reduced to 6.67 × 10^3^ (*p* = 0.01), 3.17 × 10^4^ (*p* = 0.03), and 3.08 × 10^4^ (*p* = 0.02), respectively) (Figure 1A), and 98%, 90% and 73% against A. *castellanii* 044 (the number of viable trophozoites reduced to 5.8 × 10^3^ (*p* = 0.001), 2.5 × 10^4^ (*p* = 0.002), and 7.1 × 10^4^ (*p* = 0.004),respectively) (Figure 1B), at the same test concentrations when compared to the PBS alone. In addition, chlorhexidine showed statistically significant amoebicidal activity of 62% and 53% against A. *castellanii* 044 (the number of viable trophozoites reduced to 1 × 10^5^ with *p* = 0.02, and 1.24 × 10^5^ with *p* = 0.04, respectively) (Figure 1B) at 15.6 µM and 7.81 µM in comparison to PBS alone. There was no statistically significant difference noted between the activities of the RK-758 and chlorhexidine at each test concentration against both the strains evaluated (*p* < 0.05).

### 3.2. Amoebistatic Assay

**Figure 2:**
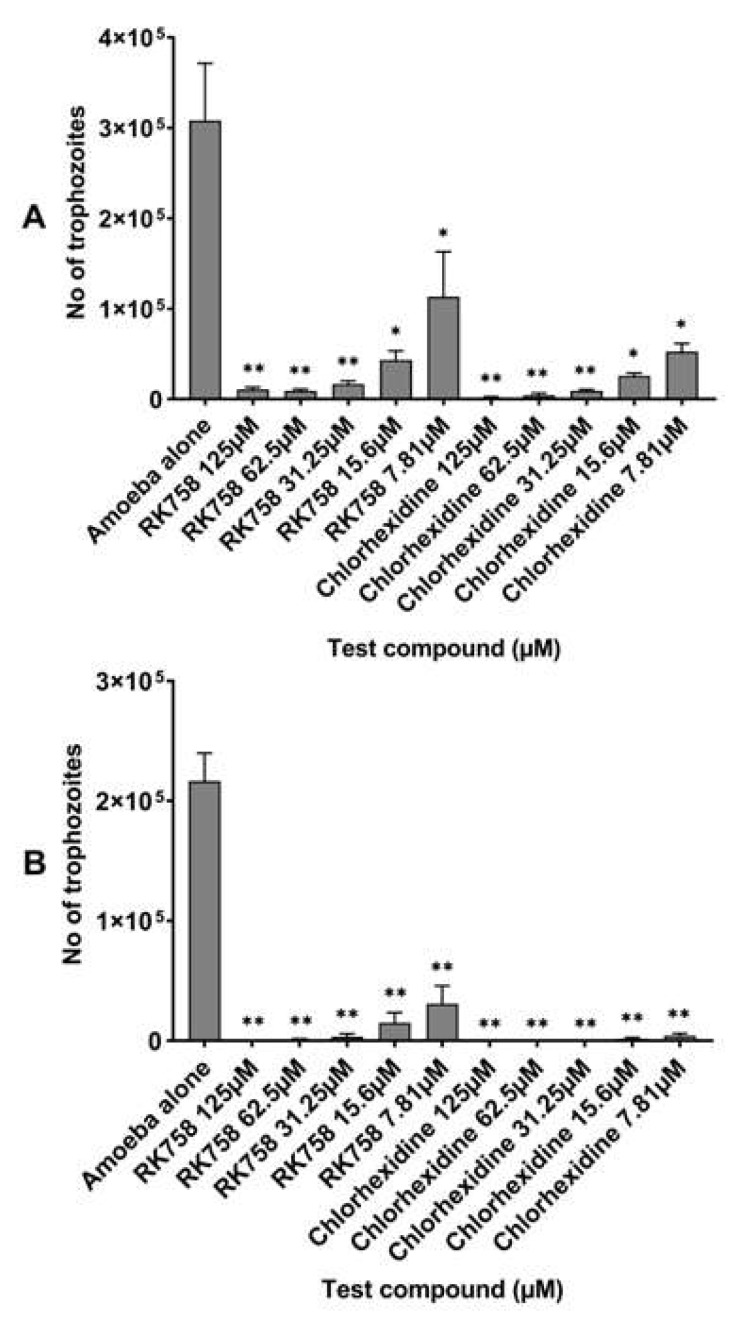
Amoebistatic activity of the peptidomimetic RK-758 in comparison with chlorhexidine against *Acanthamoeba castellanii* ATCC30868 (**A**) and *A. castellanii* 044 (**B**). In brief, 2 × 10^5^ *A. castellanii* trophozoites were incubated with the peptidomimetics RK-758 and chlorhexidine at 30 °C for 48 h after which the viability was determined by staining with Trypan blue using a Neubauer haemocytometer. The results showed significant anti-*Acanthamoeba* activity when compared to the negative control (amoeba alone). ** *p* < 0.001; * *p* < 0.05 using a two-sample *t*-test and two-tailed distribution.

Growth inhibition assays were performed to determine the amoebistatic activity of the peptidomimetic RK-758 and chlorhexidine against the *A. castellanii* ATCC30868 and 044 strains. The number of viable trophozoites was enumerated to be 3.08 × 10^5^ for *A. castellanii* ATCC30868 and 2.17 × 10^5^ for *A. castellanii* 044 following a 48 h incubation in PYG alone. The RK-758 showed the statistically significant growth inhibition of trophozoites between 97% and 63% against A. *castellanii* ATCC30868 (the number of viable trophozoites reduced to 9.17 × 10^3^ at 62.5 µM (*p* = 0.0008) and 1.13 × 10^5^ at 7.81 µM (*p* = 0.03) (Figure 2A), and 100% and 85% against A. *castellanii* 044 (the number of viable trophozoites reduced to 3.08 × 10^4^ at 7.81 µM (*p* < 0.0001) (Figure 2B), at test concentrations ranging between 125 and 7.81 µM when compared to the PYG alone. Additionally, the chlorhexidine caused a statistically significant growth inhibition against trophozoites between 99% and 83% against A. *castellanii* ATCC30868 (the number of viable trophozoites reduced to 1.67 × 10^3^ at 125 µM (*p* = 0.0007) and 5.25 × 10^4^ at 7.81 µM (*p* = 0.002) (Figure 2A), and 100% and 98% against A. *castellanii* 044 (the number of viable trophozoites reduced to 4.17 × 10^3^ at 7.81 µM, *p* < 0.001) (Figure 2B), at similar concentrations when compared to the PYG alone. There was no statistically significant difference noted between the activities of the RK-758 and chlorhexidine at each test concentration against both the strains evaluated (*p* < 0.05), except for the *A. castellanii* ATCC30868 at 125 µM, where the chlorhexidine had greater activity (*p* = 0.005).

### 3.3. Encystation Assay

**Figure 3:**
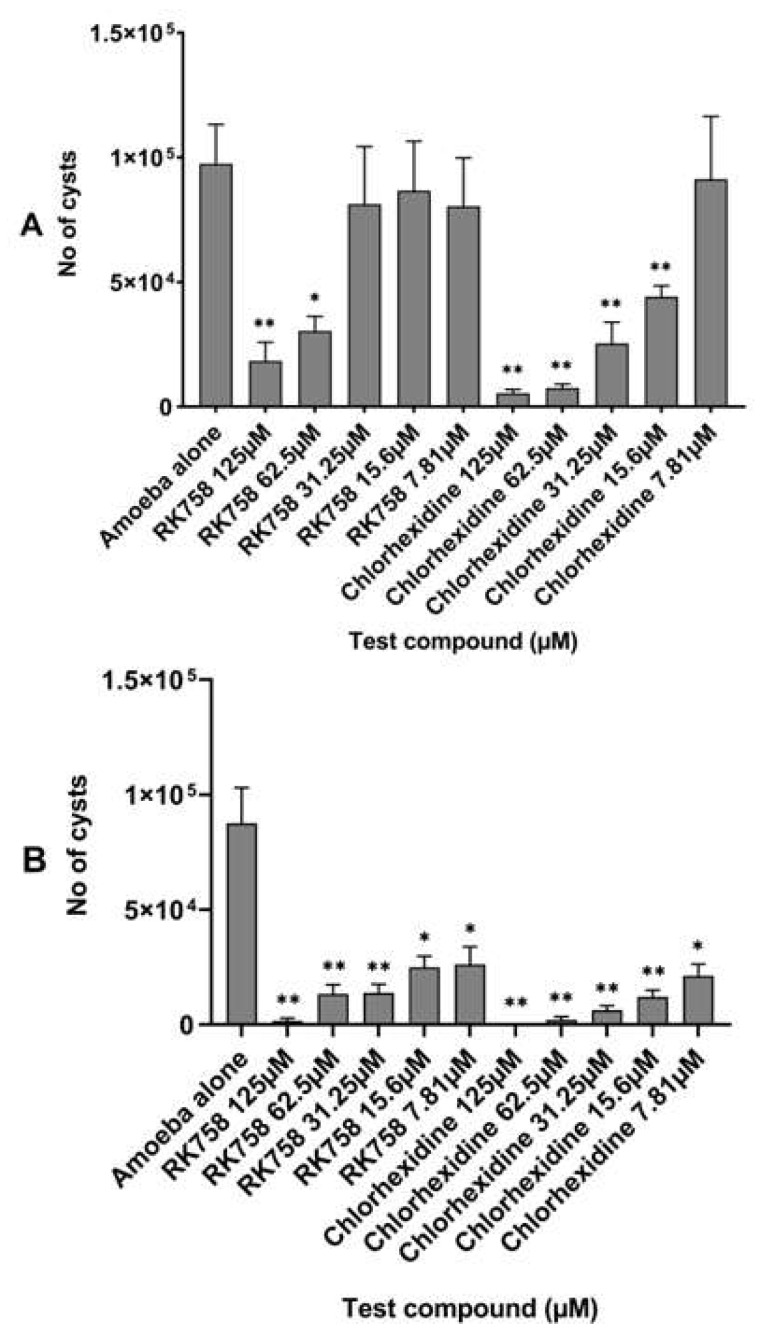
Inhibition of cysts formation by the peptidomimetic RK-758 in comparison with chlorhexidine against *Acanthamoeba castellanii* ATCC30868 (**A**) and *A. castellanii* 044 (**B**). In brief, 5 × 10^5^ *A. castellanii* trophozoites were incubated with the peptidomimetics RK-758 and chlorhexidine at 30 °C for 72 h after which cysts were determined by solubilising trophozoites adding 0.25% SDS. The number of cysts was counted using the Neubauer haemocytometer. The results showed significant anti-*Acanthamoeba* activity when compared to the negative control (amoeba alone). ** *p* < 0.001; * *p* < 0.05 using the two-sample *t*-test and two-tailed distribution.

Encystation assays were performed to determine the anti-encystation ability of the chlorhexidine and peptidomimetic RK-758 against *A. castellanii* ATCC30868 and 044 strains. At the end of the 72 h incubation, 9.75 × 10^4^ cysts of *A. castellanii* ATCC30868 and 8.75 × 10^4^ cysts of *A. castellanii* 044 had formed in the encystment medium. The RK-758 inhibited cyst formation by 81% and 68% against A. *castellanii* ATCC30868 (the number of cysts formed were 1.83 × 10^4^, *p* = 0.001, and 3 × 10^4^ with *p* = 0.002, respectively) (Figure 3A) at 125 µM and 62.5 µM test concentrations, respectively, in comparison to the encystment medium. Similarly, RK-758 inhibited cyst formation between 98% and 70% for *A. castellanii* 044 (the number of cysts formed between 1.67 × 10^3^ at 125 µM, *p* = 0.0003 and 2.63 × 10^4^ at 7.81 µM, *p* =0.005, respectively) (Figure 3B), at test concentrations ranging between 125 and 7.81 µM in comparison to the encystment medium.

Chlorhexidine inhibited cyst formation by between 94% and 54% for *A. castellanii* ATCC30868 (the number of cysts formed were 5.42 × 10^3^ at 125 µM, *p* =0.0002, and 4.42 × 10^4^ at 15.6 µM, *p* =0.008,respectively) (Figure 2A), at test concentrations ranging between 125 to 15.6 µM in comparison to the encystment medium. Additionally, chlorhexidine inhibited cyst formation between 100% and 75% against A. *castellanii* 044 (the number of cysts formed were 2.13 × 10^4^ at 7.81 µM, *p* =0.002) (Figure 2B) at test concentrations ranging from 125 to 7.81 µM in comparison to the encystment medium. There was a statistically significant difference noted between the activities of the RK-758 and chlorhexidine against the *A. castellanii* ATCC30868 at 62.5 µM and 31.25 µM concentrations (*p =* 0.003 and *p* = 0.04), respectively. Similarly, a statistically significant difference was noted between the activities of the RK-758 and chlorhexidine against the *A. castellanii* 044 at 62.5 µM and 15.6 µM concentrations (*p =* 0.02 and *p* = 0.04, respectively).

### 3.4. Excystation Assay

**Figure 4:**
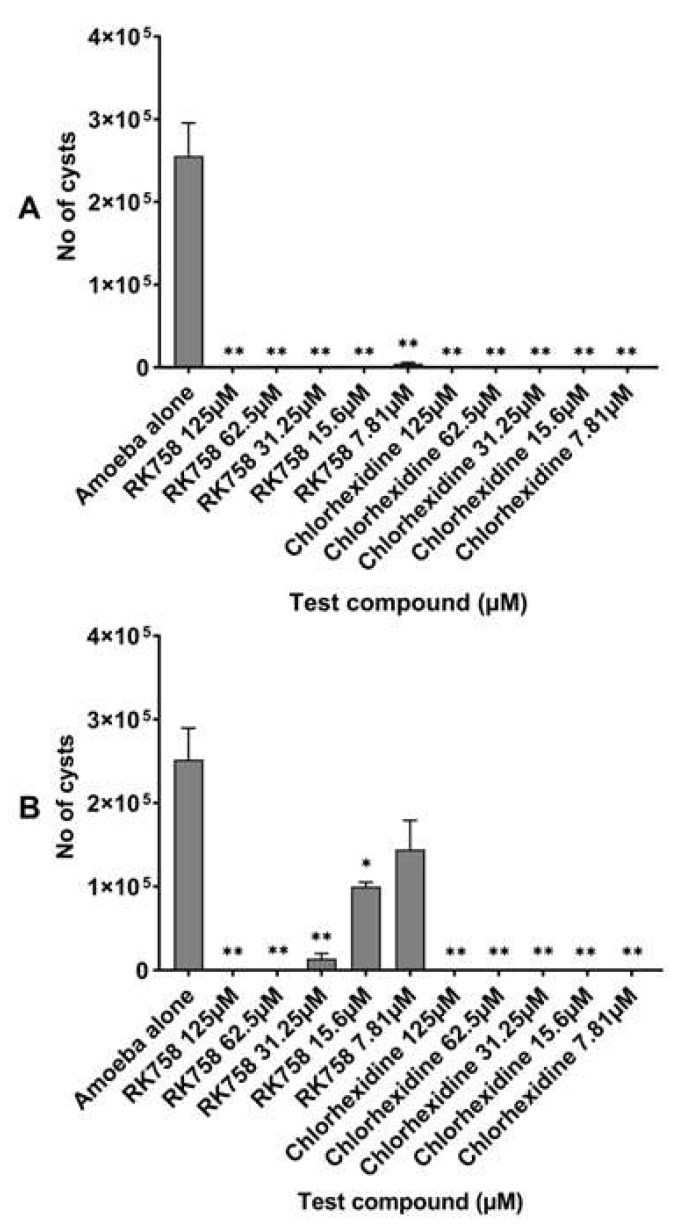
Inhibition of trophozoites’ re-emergence from cysts by the peptidomimetic RK-758 in comparison with chlorhexidine, against *Acanthamoeba castellanii* ATCC30868 (**A**) and *A. castellanii* 044 (**B**). In brief, 5 × 10^5^ *A. castellanii* cysts were incubated with the peptidomimetics RK-758 and chlorhexidine at 30 °C for 72 h after which the trophozoites were determined by counting on a Neubauer haemocytometer. The results showed significant anti-*Acanthamoeba* activity when compared to the negative control (amoeba alone). ** *p* < 0.001; * *p* < 0.05 using a two-sample *t*-test and two-tailed distribution.

**Figure 5:**
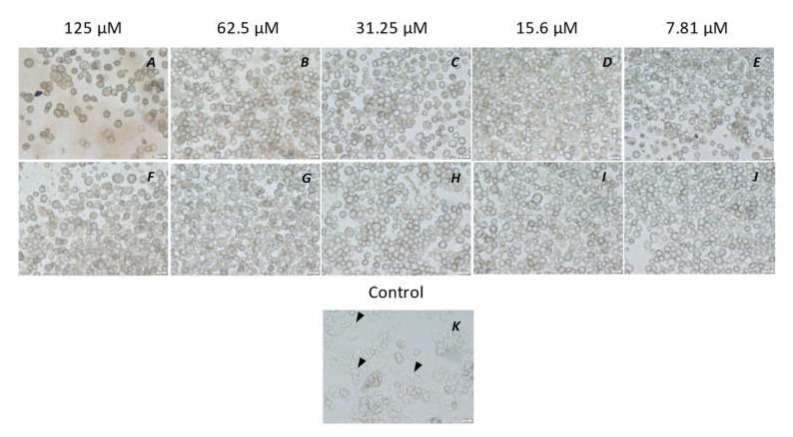
Inhibition of trophozoite emergence from cysts of *Acanthamoeba castellanii* ATCC30868 by the peptidomimetic RK-758 (**A**–**E**) in comparison with chlorhexidine (**F**–**J**), with concentrations of both ranging from 125 µM to 7.81 µM. In brief, 5 × 10^5^ *A. castellanii* trophozoites were incubated with the peptidomimetics RK-758 and chlorhexidine at 30 °C for 72 h and observed for the emergence of trophozoites. PYG alone was used as a control (**K**). PeptidomimeticsRK-758 inhibited the trophozoite emergence between concentrations of 125 µM and 31.25 µM (**A**–C) whereas the chlorhexidine inhibited their emergence between 125 µM and 7.81 µM (**F**–**J**). Arrowheads indicate trophozoites.

**Figure 6:**
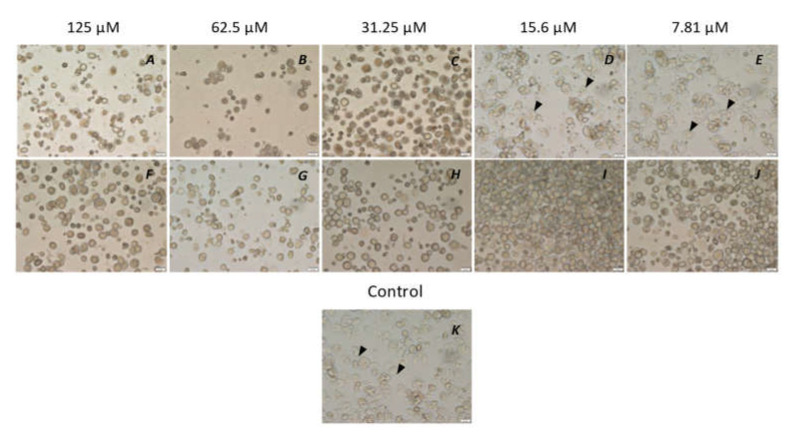
Inhibition of trophozoite emergence from cysts of *Acanthamoeba castellanii* 044 by the peptidomimetic RK-758 (**A**–**E**) in comparison with chlorhexidine (**F**–**J**), with concentrations of both ranging from 125 µM to 7.81 µM. In brief, 5 × 10^5^ *A. castellanii* trophozoites were incubated with the peptidomimetics RK-758 and chlorhexidine at 30 °C for 72 h and observed for the emergence of trophozoites. PYG alone was used as a control (**K**). Peptidomimetic RK-758 inhibited the trophozoite emergence between concentrations of 125 µM and 31.25 µM (**A**–**C**) whereas the chlorhexidine inhibited their emergence between 125 µM and 7.81 µM (**F**–**J**). Arrowheads indicate trophozoites.

Excystation assays were conducted to assess the activity of the chlorhexidine and peptidomimetic RK-758 on the re-emergence ability of trophozoites from cysts on the *A. castellanii* ATCC30868 and 044 strains. At the end of the 72 h incubation, 2.56 × 10^5^ trophozoites of *A. castellanii* ATCC30868 and 2.52 × 10^5^ trophozoites of *A. castellanii* 044 had re-emerged in the PYG medium. The peptidomimetic RK-758 inhibited the trophozoites’ re-emergence between 100% and 98% at concentrations ranging from 125 µM to 7.81 µM against *A. castellanii* ATCC30868 (the number of trophozoites excysted were 2.92 × 10^3^ at 7.81 µM (*p* = 0.0008, Figure 4A and Figure 5) in comparison to the PYG medium. Against *A. castellanii* 044, the peptidomimetic RK-758 inhibited the trophozoites’ re-emergence between 100 and 60% concentrations ranging from 125 µM to 15.6 µM concentrations (the number of trophozoites excysted were 1.00 × 10^5^ at 15.6 µM (*p* = 0.007, Figure 4B and Figure 6) in comparison to the PYG medium. Similarly, the chlorhexidine inhibited the trophozoite re-emergence by 100% at all the test concentrations between 125 µM and 7.81 µM for A. *castellanii* ATCC30868 and A. *castellanii* 044 (*p* < 0.05, Figure 4A,B) in comparison to the PYG medium.

There was a statistically significant difference between the activities of the RK-758 and chlorhexidine at 7.81 µM against A. *castellanii* ATCC30868 (*p = * 0.03) and between the activities of the RK-758 and chlorhexidine against *A. castellanii* 044 at 15.6 µM and 7.81 µM concentrations (*p* < 0.001 and *p* = 0.006, respectively).

### 3.5. Haemolysis Assay

Chlorhexidine and peptidomimetic RK-758 caused haemolysis in a dose-dependent manner. Chlorhexidine showed a negligible haemolysis between 31.25 µM and 7. 81 µM. The therapeutic index (i.e., haemolytic concentration/antimicrobial concentration) for the chlorhexidine ranged from 16 to 4. The peptidomimetic RK-758 showed haemolysis of < 50% between 31.25 µM and 7.81 µM. The therapeutic index of the RK-758 ranged from 8 to 1.

## 4. Discussion

This study demonstrated that the peptidomimetic RK-758 was active against both the trophozoite and cyst forms of *Acanthamoeba castellanii*, most probably due to its cationic charge [19,20,25]. The anti-amoebic effects of any test compounds on both trophozoites and cysts are crucial as the trophozoites can remerge and lead to a relapse of the disease upon stopping treatment [1,4,5]. This may possibly be due to the risk of cysts’ formation in corneal tissue during *Acanthamoeba* infections [51]. A laboratory study examining the in vitro sensitivity of 23 isolates from 23 patients to 13 different drugs found that PHMB and chlorhexidine were the most successful agents against both trophozoites and cysts [52]. Recently, a retrospective study showed that treatment with biguanides and diamidines resulted in a 64% improvement in the best-corrected visual acuity in patients [5]. A feature that is commonly seen in AK is an initial worsening of inflammation upon starting treatment, and this may possibly be due to the antigens released by the dead organisms [8,53]. The current results show that the peptidomimetic RK-758 possessed amoebicidal and cysticidal activity against both the ATCC and the clinical strain tested, which is essential in tackling an *Acanthamoeba* infection.

Previous studies have already established that peptidomimetics are highly effective against bacteria [35,36,37], but before this study, the anti-*Acanthamoeba* effects of peptidomimetics had not been evaluated.

Previously, the naturally occurring antimicrobial peptide protamine had been shown to have amoebicidal activity at 228 µM, and its derivative melimine inhibited *Acanthamoeba* trophozoites’ adhesion to contact lens at a concentration of 152 µg per lens [10,19]. Additionally, another naturally occurring antimicrobial peptide magainin had minimal inhibitory (90% reduction) and minimal amoebicidal (99.9% reduction) activities between 8 and 16 µM [20,21]. The antimicrobial peptide poly-epsilon-lysine was amoebicidal to trophozoites and cysts at 540 µM producing an 80% death of trophozoites and 76% death of cysts at the end of a 24 h incubation [25]. An AMP derived from tyrocidine, when used at 100 µg/mL and 250 µg/mL, killed 35% and 84% of trophozoites, respectively, at the end of a 24 h incubation and reduced the transformation of trophozoites to cysts by 58% and 93%, respectively, at the end of a 72 h incubation [26]. The exposure of *A. castellanii* trophozoites to nisin, a bacteriocin AMP, resulted in reductions in cultivable numbers after a 24 h exposure but there was a recovery in the amoeba growth after a 72 h exposure. This may have been due to the lack of a nisin effect on the membrane integrity of the trophozoites [28]. A synthetic decapeptide AMP (called KP) killed 57% of trophozoites at 25 µg/mL after a 24 h incubation [27]. The peptidomimetic RK-758 was effective between 7.81 µM and 62.5 µM against both the strains evaluated. This was somewhat less effective than reported for magainin, but was improved over the reports for protamine and poly-epsilon-lysine. Initial proteolysis assays with trypsin (unpublished) showed that RK-758 was proteolytically stable for 18 h, possibly due to its substituted guanidine end cap. Magainin, however, can be cleaved by proteases [54], which may be one of the reasons it did not outperform the antibiotic ofloxacin in a Phase III clinical trial against bacteria [55].

The current study found that RK-758 performed almost equivalently to the currently used topical treatment, chlorhexidine. Chlorhexidine has previously been reported to have minimum cysticidal activity against nine *Acanthamoeba* clinical isolates at 3.1 to 25 µg/mL (6 to 49 µM) [56] and the current study showed similar results against both trophozoites and cysts; however, chlorhexidine has been reported to be toxic to the eyes causing corneal irritation and abrasions [57]. Future studies should determine the ocular safety of RK-758 to determine if it has an improved ocular safety profile over chlorhexidine.

A haemolysis assay is often used as a primary measure of the toxicity of antimicrobial peptides [35]. The RK-758 produced minimal haemolysis at 15.6 µM and 7.81 µM, giving a therapeutic index of up to eight. Using LD_50_ (a dose causing 50% death in mice) as a measure of toxicity, polyhexamethylene biguanide, another commonly used therapeutic for *Acanthamoeba* keratitis [8,58], has a therapeutic index of 3.2 [59]. However, haemolysis and LD_50_ are not directly comparable; therefore, experiments directly comparing the therapeutic index of currently used therapies with RK-758 are required. A previous study showed that chlorhexidine at 20 µg/mL (31.97 µM) gave a 20% lysis of rabbit red blood cells after 2 h of incubation [60]. Similarly, the current results showed a negligible lysis of horse red blood cells at a similar concentration (31.25 µM) and a higher concentration (62.5 µM) after 4 h of incubation.

The current study used PBS as a negative control to evaluate the viability of trophozoites in an amoebicidal assay; however, a previous study reported that PBS alone induced 28% encystment after 24 h of incubation [41]. A later study showed only a 4% encystment at the end of a 24 h incubation and no effect on the viability of *A. castellanii*. The authors suggested that the inclusion of 50 mM of MgCl_2_ and 10% glucose in the PBS formulation induced encystment [48]. Elsewhere, PBS solutions containing 0.25 ppm PHMB and increasing concentrations of propylene glycol and povidone stimulated encystment in a dose-dependent manner [61]. The current study did not find any encystment during the evaluation of the trophozoite viability.

In summary, the peptidomimetic RK-758 has excellent anti-*Acanthamoeba* activity on the trophozoites and cysts of *A. castellanii* ATCC30868 and 044 strains. Its activity is very similar to chlorhexidine, which indicates that RK-758 has the potential to be developed as a new therapeutic agent for the treatment of *Acanthamoeba* keratitis or a new disinfectant for contact lenses with good activity against *Acanthamoeba*.

## Data Availability

The data supporting the conclusions of this article are included within the article and its figures. No large datasets were generated or analysed during the current study.
